# Recurrent transmural tracheal schwannoma resected by video-assisted thoracoscopic window resection: A case report

**DOI:** 10.1097/MD.0000000000018180

**Published:** 2019-12-20

**Authors:** Huiguo Chen, Kai Zhang, Mingjun Bai, Haifeng Li, Jian Zhang, Lijia Gu, Weibin Wu

**Affiliations:** a Department of Cardiothoracic Surgery, the Third Affiliated Hospital, Sun Yat-sen University; b Department of Vascular Interventional Radiology, the Third Affiliated Hospital, Sun Yat-sen University; c Department of Pathology, the Third Affiliated Hospital, Sun Yat-sen University, Guangzhou, Guangdong, People's Republic of China.

**Keywords:** endoscopic excision, tracheal schwannoma, tumor recurrence, video-assisted thoracoscopic surgery, window resection

## Abstract

**Rationale:**

Primary schwannoma is extremely rare in the trachea, and its optimal treatment has not yet been established. Previous literature have indicated that traditional resection by thoracotomy is an effective surgical procedure but with huge trauma, and endoscopic excision is a minimally invasive surgical method but with possibility of recurrence. Window resection was usually utilized for selected patients with trachea invasion by thyroid carcinoma, but video-assisted thoracoscopic window resection for trachea schwannoma has not been reported previously.

**Patient concerns:**

A 23-year-old woman was admitted to hospital due to dyspnea, coughing and wheezing that had persisted for 2 months with aggravation for 1 week.

**Diagnoses:**

Chest computed tomography (CT) scan revealed a well-circumscribed soft-tissue mass located on the right lateral posterior wall of the trachea. Bronchofibroscopy (BFS) showed a whitish, smooth and round mass with a wide base in the trachea. Immunohistochemical staining demonstrated cells labeled with Vim (+), S-100 (+), SOX-10 (+), SMA (–), CK (–). Histopathological examinations showed that the mass was a schwannoma.

**Interventions:**

The tumor was nearly completely excised via BFS, but relapsed 2 times at 12 days and 3 weeks after endoscopic resection. Finally, the patient underwent video-assisted thoracoscopic window resection of trachea.

**Outcomes:**

The patient recovered rapidly and no recurrence was observed over 6 months of follow-up.

**Lessons:**

The treatment of tracheal schwannoma depends on the characteristics of tumor and the condition of patient. Surgical resection is a preferred alternative for sessile or transmural tumors and recurrence after endoscopic excision. Tracheal window resection by video-assisted thoracoscopy is beneficial for some appropriate patients with a small and sessile tumor.

## 1 Introduction

Schwannomas originate from Schwann cells and they are benign lesions usually. Primary schwannoma is extremely rare in the trachea being more frequently reported in the lungs and bronchi and the exact frequency is unknown.^[1]^ Thus far, the data of primary tracheal schwannoma available is limited, its optimal treatment has not yet been established. Previous literature have indicated surgical resection and endoscopic excision have been successfully utilized in the treatment of this disease,^[2,3]^ especially endoscopic surgery is widely used with the advance of equipment and technology. It is undeniable that endoscopic surgery has the advantages of less trauma and faster recovery, but the possibility of local recurrence should not be ignored.^[1–4]^ Circumferential resection and end-to-end anastomosis of the trachea by thoracotomy is usually adopted in the treatment of tracheal schwannoma,^[1,2]^ but the surgery is hugely traumatic and risky, and may lead to severe complications and bilateral recurrent laryngeal nerve injury. Window resection was usually used for selected patients with trachea invasion by thyroid carcinoma with favorable outcomes,^[5,6]^ but it has not been reported in the treatment of tracheal schwannoma so far. Therefore, we attempted to resect a primary tracheal schwannoma, following recurrence after endoscopic excision two times, by video-assisted thoracoscopic window resection. In addition, we emphasize the selection of treatment along with a literature review.

## 2 Methods

The informed consent for publication of this case report and images were obtained from the patient. Since the personal information and images presented in the article are entirely unidentifiable, the Ethics Committee of our institution waived the requirement for ethical approval.

## 3 Case presentation

**Figure 1:**
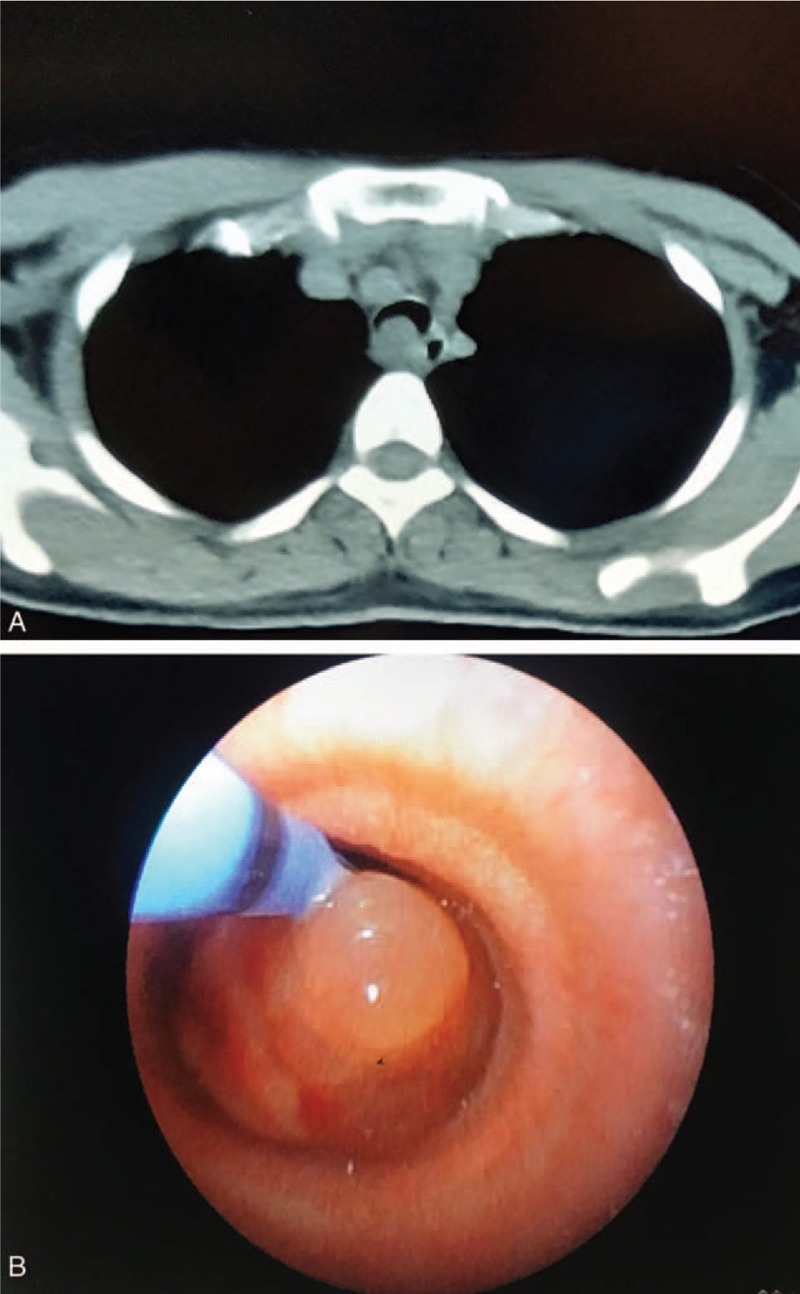
Intratracheal schwannoma on CT scan and bronchofibroscopy examination before first endoscopic surgery. A: Chest CT showing a ∼1.5 cm intratracheal tumor (arrow), located on the right lateral posterior wall of the trachea at the level of the brachiocephalic veins. B: Bronchofibroscopy revealing a whitish, round mass with a wide base, nearly obstructing about 90% of the tracheal lumen.

**Figure 2:**
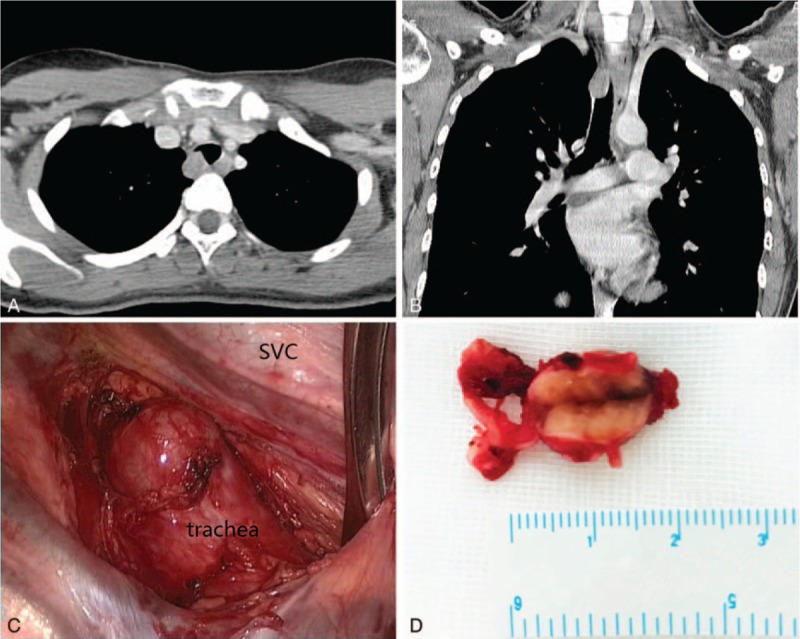
Intratracheal schwannoma on CT scan and intraoperative findings. A and B: Enhanced and 3-dimensional reconstructive CT indicated the tumor with an irregular shape, obscure boundary and moderate enhancement, and protruded into the right thoracic cavity obviously. C and D: Intraoperative findings: The tumor had an integrated capsule with several small vessels (arrow). The cut surface revealed a pale-yellow appearance, partially intermingled with brown. SVC = superior vena cava.

**Figure 3:**
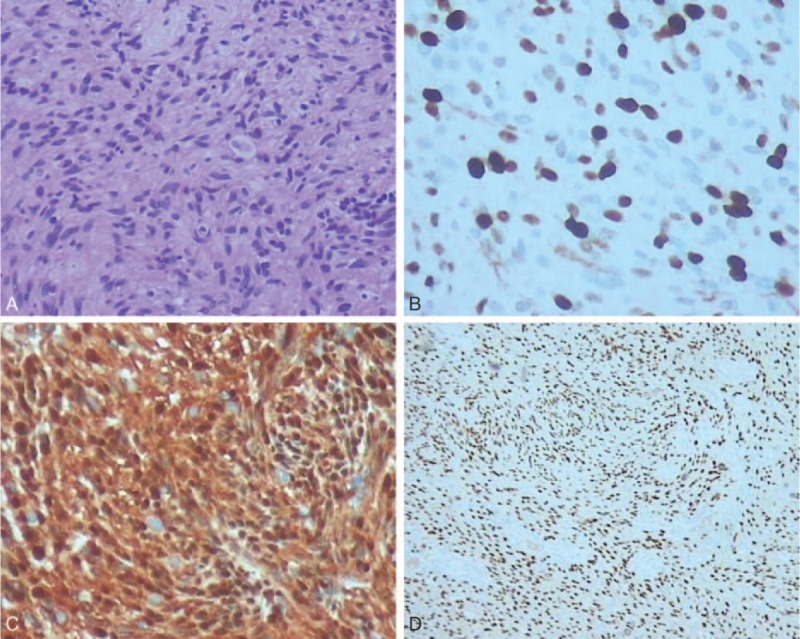
Pathological findings. A: Hematoxylin and eosin staining showed spindled cells and elongated nuclei arranged in a palisading pattern (200 × magnification). B and C: Immunohistochemical staining showed strong positivity for SOX-10 and S-100 (200 × and 40 × magnification, respectively). D: Ki-67 index was ∼ 20% (200 × magnification).

A 23-year-old woman was admitted to department of respiratory and intensive care unit on September 29th, 2018 due to dyspnea, coughing and wheezing that had persisted for 2 months with aggravation for 1 week obviously. The patient denied chest pain, fever and palpitation. Physical examination revealed a temperature of 36.4°C, a heart rate of 80 to 90 beats/minute, a blood pressure of 113/76 mmHg, a respiratory rate of 16 to 20 breaths/minute and a transcutaneous oxygen saturation 99% on room air. Lung auscultation showed wheezing in the both inspiratory and expiratory phases and a prolonged expiratory phase on the cervical trachea. In the Outpatient Clinic, the chest x-ray did not show any abnormality 1 month ago, but the following chest plain computed tomography (CT) scan revealed a ∼1.5 cm, well-circumscribed soft-tissue mass located on the right lateral posterior wall of the trachea at the level of the brachiocephalic veins (Fig. 1A). Bronchofibroscopy (BFS) showed a whitish, smooth and round mass with a wide base, locating at the 3 to 7 o’clock position of the trachea about 6 cm below the vocal cords, and nearly obstructing about 90% of the tracheal lumen (Fig. 1B). The tumor was nearly completely excised by argon plasma coagulation (APC) combined with cryotherapy via BFS(Fig. 1B), and the symptoms were alleviated after the treatment immediately. Morphologically, hematoxylin and eosin staining showed the tumor comprised compact bundles of spindle cells with elongated palisading nuclei, fibrillary cytoplasm and rare mitotic figures (Antoni A pattern). Immunohistochemical analysis showed that the tumor was positive for vimentin, S-100, SOX-10 (SRY -Box 10) and negative for smooth muscle actin (SMA), desmin, cytokeratin (CK), CD34, and the ki-67 index was about 3%. Finally, the pathology confirmed the mass to be a benign schwannoma. However, the patient suffered from dyspnea again 12 days later. BFS found the tumor recurred and obstructed more than 90% of the tracheal lumen. Electrosurgical snaring combined with protractor biopsy was used to cut the mass under BFS. Unfortunately, serious dyspnea and cough recurred 3 weeks later. Chest plain CT and bronchofibroscopic examination confirmed the tumor relapsed. Immediately, the patient was received the tumor resection via BFS once again. Then the patient was admitted to our hospital on November 12th, 2018. Chest contrast-enhanced CT indicated the tumor relapsed in situ with an irregular shape, obscure boundary and moderate enhancement, about one third of the tracheal circumference and protruded into the right thoracic cavity obviously (Fig. 2A and B). Immediately, the patient underwent video-assisted thoracoscopic window resection of trachea and longitudinal suture with uneventful recovery. Macrography, the tumor was about 16 × 14 × 10 mm in size with an integrated smooth capsule (Fig. 2C and D). Frozen pathology showed benign tumor and margins were free of tumor. The pathologic findings were consistent with previous result (Antoni A pattern, positive for SOX-10 and S-100) (Fig. 3A–C) except more mitotic figures and the ki-67 index was up to 20% (Fig. 3D). Finally, a diagnosis of benign schwannoma with focal of actively proliferated cells was made. The patient discharged from hospital 7 days after operation. There was no recurrence after 6 months follow-up by chest CT and BFS.

## 4 Literature review and discussion

Schwannoma is a benign tumor arising from Schwann cells of the peripheral nerve, and it usually occurs in the head, neck, retroperitoneum and extremities. The tumor is more frequently reported in lungs and bronchi but not trachea in respiratory system.^[1]^ Primary tracheal schwannoma is extremely rare, and it was first reported by Straus et al in 1951.^[7]^ We did computerized the English literature (abstract or full test) searches of the PubMed, there were only 51 cases of primary tracheal schwannoma were identified from 1950 to 2013,^[1]^ and after that to October 2018, only 9 cases had been reported in addition.^[1,8–15]^

Because of the rarity of tracheal schwannoma, its optimal treatment has not yet been established. In the published literature,^[9,14,16]^ the therapeutic method of tracheal schwannoma includes surgical resection and endoscopic excision based on the size, the location and the extension of the tumor. According to the tumor's site and extension, Kasahara et al^[17]^ proposed a classification of the pulmonary schwannomas. They divided the pulmonary schwannomas into 2 types: central type and peripheral type. According to the relationship between tumor and tracheal luminal space, the central type is classified in to the following 2 subtypes:

(1) intraluminal type which tumors exist only in the intraluminal space and
(2) transmural type which tumors occur in both intraluminal and extraluminal spaces (Table 1).

**Table 1:**
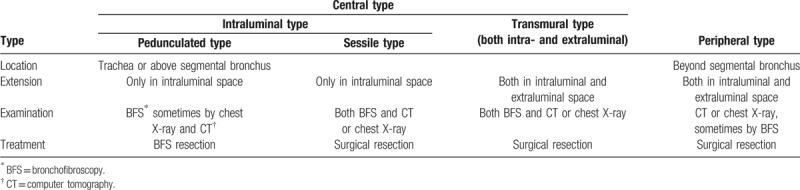
Classification of pulmonary schwannomas according to its location and extension.

The treatment for primary tracheal schwannoma should consider the characteristics of tumor and the condition of patient. In the published literature, surgical resection and endoscopic excision are utilized in the treatment of tracheal schwannoma mainly.^[1–4]^ In these 4 excellent case reports with literature review, about 30% patients were underwent endoscopic excision and 70% patients were underwent surgical resection by traditional thoracotomy. For tumors with pedicle and only in intraluminal space (pedunculated type),^[17,18]^ and in those patients with surgical contraindications or in emergency situation due to severe obstruction by tumor, endoscopic excision may be an appropriate option for radical resection or debulking surgery relieving airway obstruction. For tumors with broader base (sessile type) or both intraluminal and extraluminal (transmural type), and in those patients without surgical contraindications, surgical resection is a preferred alternative.^[11]^ However, there were some recurrent cases with endoscopic treatment (the recurrence rate from 4% to 21%),^[1,3]^ while there was no recurrent case with surgical resection in these reports. The results indicated that endoscopic treatment had the possibility of local recurrence, therefore, careful selection of treatment for appropriate patients should be considered for reducing the risk of recurrence. Recurrent case after endoscopic excision was strongly recommended surgical resection rather than endoscopic treatment repeatedly.^[1,11]^

In the present patient, a transmural tumor both in intraluminal and extraluminal space of trachea was detected by CT initially, and it was confirmed and partial resected via BFS to relief severe airway obstruction. It was seen to be a reasonable treatment option due to the urgent condition. According to the above principles, in such a case with high risk of recurrence, surgical resection of the residual tumor should be planned in time. Unfortunately, the patient was not to be advised surgical resection. Consequentially, the tumor recurred 2 times, respectively, in 12 days and 3 weeks later after endoscopic excision. Finally, the patient was admitted to our hospital and received surgical resection without relapse in 6 months of follow-up.

Tracheal resection and end-to-end anastomosis by traditional thoracotomy are usually adopted in tracheal schwannoma,^[1,11]^ but this approach is a high-risk surgery due to intraoperative transthoracic tracheal intubation, difficult tracheal end-to-end anastomosis and huge trauma, and it may lead to recurrent laryngeal nerve injury. Window resection was usually used for trachea invasion by thyroid carcinoma with good outcomes,^[5,6]^ and the above problems were overcome. This is useful if tumor invasion is less than one quarter of the tracheal circumference and <2 cm in its vertical dimension.^[5]^ In this case, though the tumor occupied about one third of the tracheal circumference, but it was a benign tumor and only 1.4 cm in its vertical dimension. Therefore, we attempted to performe video-assisted thoracoscopic tracheal window resection and longitudinal suture in this case and succeeded. Preliminary identification of this method could resect a small tracheal schwannoma completely and safely. Compared to circumferential resection by traditional thoracotomy, this surgical procedure overcomes intraoperative transthoracic intubation and reduces the trauma and the risk of surgery. In addition, the patient did not need to maintain neck flexion and recover quickly. But this approach has some limitations. For example, it is not suitable for the large tumor or the tumor involved carina. We consider that the surgery approach is suitable for the tracheal tumors which occupy less than one third of the tracheal circumference and <1.5 cm in its vertical dimension above the carina. Long-time follow-up is required to evaluate outcomes and complications, such as recurrence and airway stenosis. And this approach needs to be further explored by studies of larger patient samples.

Meanwhile, we found the pathology had more mitotic figures and the ki-67 index raised from 3% to 20% after endoscopic treatment repeatedly. Though malignant transformation of schwannoma after APC, cryotherapy or electrosurgical snaring has not been reported, but there were some cases about malignant transformation of vestibular schwannoma spontaneously or after radiation.^[19–21]^ So, whether malignant transformation of tracheal schwannoma after stimulation (such as APC, cryotherapy et al), long-time follow-up is needed and surgical resection is recommended after endoscopic excision with recurrence.

## 5 Conclusion

Primary tracheal schwannoma is extremely rare. The treatment depends on the characteristics of tumor and the condition of patient. Surgical resection is a preferred alternative for sessile or transmural tumors and recurrence after endoscopic excision. Tracheal window resection by video-assisted thoracoscopy is beneficial for some appropriate patients with a small and sessile tumor, but long-term follow-up is required.

## Author contributions

**Conceptualization:** Huihuo Chen, Weibin Wu.

**Data curation:** Huihuo Chen, Kai Zhang, Mingjun Bai, Lijia Gu.

**Visualization:** Mingjun Bai, Haifeng Li, Jian Zhang.

**Writing – original draft:** Huihuo Chen, Jian Zhang.

**Writing – review & editing:** Kai Zhang, Weibin Wu.
